# Using BOX-PCR to exclude a clonal outbreak of melioidosis

**DOI:** 10.1186/1471-2334-7-68

**Published:** 2007-06-30

**Authors:** Bart J Currie, Daniel Gal, Mark Mayo, Linda Ward, Daniel Godoy, Brian G Spratt, John J LiPuma

**Affiliations:** 1 Northern Territory Clinical School, Flinders University, Royal Darwin Hospital, Darwin, Northern Territory, Australia; 2 Tropical and Emerging Infectious Diseases Division, Menzies School of Health Research, Charles Darwin University, Darwin, Northern Territory, Australia; 3 Department of Infectious Diseases Epidemiology, Faculty of Medicine, Imperial College London, St. Mary's Hospital, London, UK; 4 Department of Pediatrics and Communicable Diseases, University of Michigan Medical School, Ann Arbor, Michigan, USA

## Abstract

**Background:**

Although melioidosis in endemic regions is usually caused by a diverse range of *Burkholderia pseudomallei *strains, clonal outbreaks from contaminated potable water have been described. Furthermore *B. pseudomallei *is classified as a CDC Group B bioterrorism agent. Ribotyping, pulsed-field gel electrophoresis (PFGE) and multilocus sequence typing (MLST) have been used to identify genetically related *B. pseudomallei *isolates, but they are time consuming and technically challenging for many laboratories.

**Methods:**

We have adapted repetitive sequence typing using a BOX A1R primer for typing *B. pseudomallei *and compared BOX-PCR fingerprinting results on a wide range of well-characterized *B. pseudomallei *isolates with MLST and PFGE performed on the same isolates.

**Results:**

BOX-PCR typing compared favourably with MLST and PFGE performed on the same isolates, both discriminating between the majority of multilocus sequence types and showing relatedness between epidemiologically linked isolates from various outbreak clusters.

**Conclusion:**

Our results suggest that BOX-PCR can be used to exclude a clonal outbreak of melioidosis within 10 hours of receiving the bacterial strains.

## Background

While melioidosis is well recognised as endemic to southeast Asia and northern Australia, the geographical range of environmental presence remains poorly defined for the causative bacterium,*Burkholderia pseudomallei*[1,2]. Cases of locally-acquired melioidosis have recently been reported from Brazil[3], Honduras[4] and Taiwan[5].

In endemic regions molecular typing of *B. pseudomallei *has shown considerable genetic diversity. For example, in northern Australia, although cases cluster in the monsoonal wet season, isolates from individual patients are usually distinct from each other[6]. An exception is when cases can be epidemiologically linked to a point source outbreak such as contamination of a community water supply[7,8]. There are major public health implications if a series of melioidosis cases is found to be clonal in nature and therefore a possible point source outbreak. *B. pseudomallei *is classified as a CDC Group B bioterrorism agent and the ability to quickly distinguish endemic infection from a clonal cluster has been problematic because of the time needed to perform the molecular typing with the methods commonly used to date.

We have therefore adapted BOX-PCR for typing *B. pseudomallei *and compare BOX typing results on a wide range of well-characterized *B. pseudomallei *isolates with multilocus sequence typing (MLST) and pulsed-field gel electrophoresis (PFGE).

## Methods

### BOX-PCR

Repetitive sequence PCR using a BOX A1R primer (BOX-PCR fingerprinting) was adapted from the methods used by Coenye et al. for *B. cenocepacia*[9]. Single bacterial colonies were subcultured overnight at 37°C on chocolate agar (Oxoid Australia, Melbourne, Australia). Bacterial DNA was purified with the DNeasy Tissue Kit (Qiagen, Hilden, Germany) using the Gram-positive bacteria protocol but with only 1 mg/mL lysozyme and 45 min incubation with Proteinase K at 55°C. Each BOX-PCR (25 μL) contained template DNA (1.5 ng); BOX-A1R primer (0.3 μg); 600 μM dNTP (Roche, Basel, Switzerland); 2 U HotStarTaq Plus (Qiagen); 1× Q-Solution (Qiagen) and 1× PCR buffer (Qiagen) with 6.0 mM MgCl_2_. The PCR was first denatured at 95°C for 5 min; followed by 10 cycles of denaturing at 94°C for 10 s, annealing at 52°C for 1 min, elongating at 68°C for 4 min; then for the next 25 cycles, the elongation step is extended by 10 s for each additional cycle. Amplicons were separated by gel electrophoresis on 2% agarose (1× TAE buffer).

### Pulsed-field gel electrophoresis and multilocus sequence typing

PFGE (*Spe *I) and MLST were performed as previously described[10,11].

### Data analysis

For BOX-PCR and PFGE, gel images were analysed with BioNumerics (version 4.50; Applied Maths BVBA, Sint-Martens-Latem, Belgium). The BioNumerics application modules used were the Fingerprint types and the Comparison and Cluster Analysis modules. BOX-PCR bands (200–1500 bp) were auto-detected by BioNumerics set to 10% minimum profiling relative to maximum value of lane and 5% minimum area. PFGE bands (150–700 kbp) were manually assigned on visual inspection. Both BOX-PCR and PFGE dendrograms were produced with Dice UPGMA with position tolerance settings of 0.4% optimization, 0.8% band position tolerance and 0.1% change towards end of fingerprint.

For MLST the alleles at each of the seven previously described loci[11] were assigned for each isolate by comparing the sequences to those at the *B. pseudomallei *MLST website[12]. Following the standard MLST protocol, each allele is assigned a different allele number and the allelic profile (string of seven integers) is used to define the sequenced type (ST) for that isolate. The relatedness of isolates was displayed as a dendrogram by using the matrix of pairwise differences in the allelic profiles of the isolates and the unweighted pair group method with arithmetic averages (UPGMA), using *MEGA *version 3.1[13].

### *B. pseudomallei *isolates

To assess the discriminatory power of BOX-PCR, direct comparisons were made between the MLST dendrogram for 54 separate STs and the BOX-PCR dendrogram for these isolates. The 54 *B. pseudomallei*, each with a distinct ST, were all from Australia and included human, animal and environmental isolates. Amongst these were 11 pairs of single locus variants (SLVs; two isolates sharing identical alleles at 6/7 loci).

To assess the ability of BOX-PCR to identify clonal clusters direct comparisons were then made between the PFGE dendrogram for five defined clonal groups and the BOX-PCR dendrogram for these isolates. Clonal cluster I and clonal cluster II consist of 7 and 8 isolates, respectively, from Australia's tropical Northern Territory and were previously identified as clustering by PFGE[14]. These two clonal clusters represent geographically linked but epidemiologically unrelated isolates from our prospective melioidosis studies in northern Australia. Clonal cluster III consists of 3 isolates of identical ST from a detergent container implicated in an outbreak of melioidosis in the Northern Territory involving two garage mechanics[10]. Clonal cluster IV consists of 3 isolates from an outbreak of melioidosis involving hobby farms in a temperate location in southwest Western Australia. This outbreak spanned 25 years and was attributed to possible importation of an infected animal into a region not endemic for melioidosis[15,16]. Clonal cluster V is 6 isolates from an outbreak of melioidosis in a remote Northern Territory indigenous community. The outbreak was linked to contamination of the unchlorinated community water supply, with several deaths reported[8].

## Results

**Figure 1:**
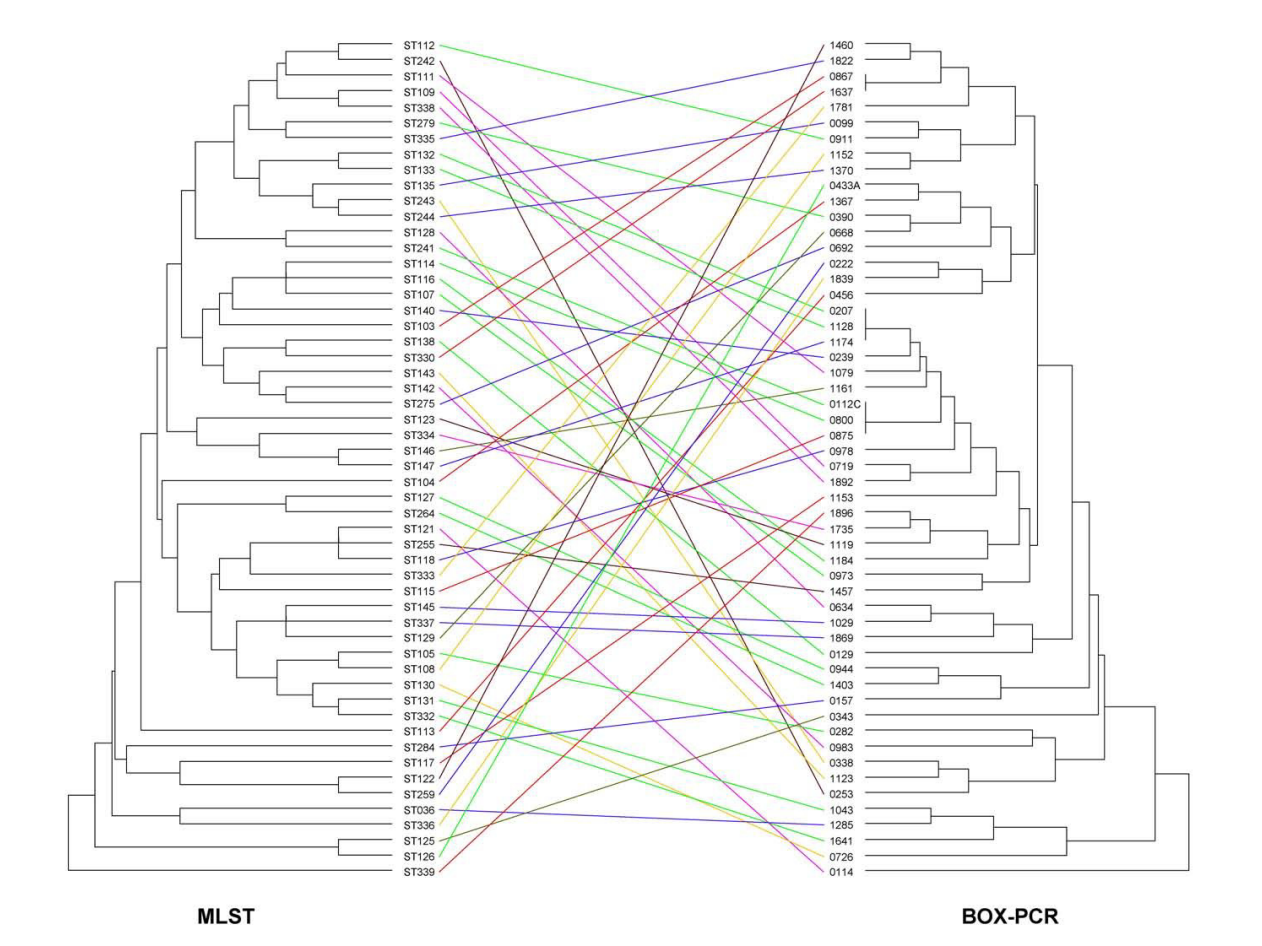
Comparison of MLST and BOX-PCR dendrograms for 54 *B. pseudomallei *isolates. The MLST sequence type (ST) is shown for each isolate, with the corresponding isolate number listed for the BOX-PCR profile.

Figure 1 shows the relationship between the 54 discrete MLST STs and the BOX-PCR for these isolates. Except for 1 pair of STs and 2 triplets, BOX-PCR was able to discriminate between each ST. While relationships between STs seen on the MLST dendrogram were not consistently preserved with BOX-PCR, there was some clustering as evident from Figure 1, especially for some SLVs and double locus variants (two isolates sharing identical alleles at 5/7 loci).

**Figure 2:**
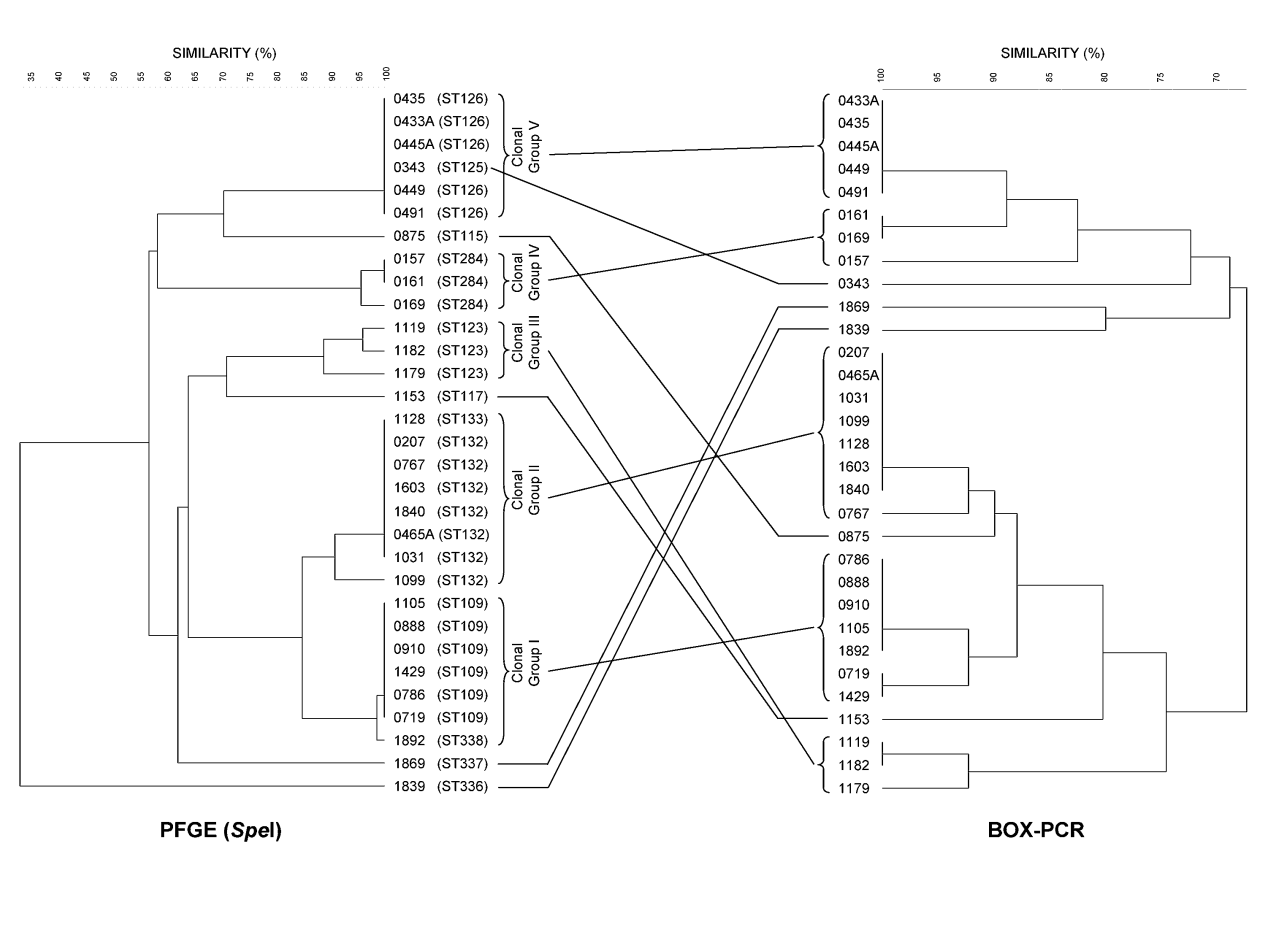
Comparison of PFGE and BOX-PCR for isolates in 5 clonal groups (see text for details). Isolate number with its MLST ST is listed for each isolate on the PFGE profile, with the corresponding isolate number listed for the BOX-PCR profile. Four unrelated isolates are included for comparison; 0875 (ST 115), 1153 (ST 117), 1869 (ST 337) and 1839 (ST 336). Clonal Group I includes ST 109 and ST 338; Clonal Group II includes ST 132 and ST 133; Clonal Group III is ST 123; Clonal Group IV is ST 284 and Clonal Group V includes ST 125 and ST 126.

Figure 2 shows results for the 27 isolates in the cluster study, represented by 8 STs within the 5 clonal groups, with 4 additional unlinked isolates each from a different ST included for comparison. There was generally excellent agreement between PFGE and BOX-PCR for each of the five clonal clusters. Of interest, BOX-PCR split isolate 0343 from other isolates in PFGE cluster V. Isolate 0343 is indeed slightly different by MLST, being a SLV (ST 125) of the other 4 patient isolates and the water isolate (isolate 491, ST 126) from this outbreak. In PFGE cluster II isolate 1128 was a SLV (ST 133) of the other 7 isolates (ST 132), but both PFGE and BOX-PCR did not split these. However isolate 0767 (ST 132) in this cluster was slightly split from the other ST 132 isolates on BOX-PCR. In cluster III both PFGE and BOX-PCR identified isolate 1179 as being split from 1119 and 1182, although all three are ST 123. In clonal clusters I and IV there were similar small variations between PFGE, MLST and BOX-PCR.

## Discussion

Various methods have been developed for typing strains of *B. pseudomallei*. Ribotyping was the earliest method widely used[17], including the first report where genetic fingerprinting was able to link human and animal *B. pseudomallei *isolates to an environmental isolate from contaminated farm soil[16]. However, ribotyping is cumbersome and costly. Although PFGE has largely replaced ribotyping, it also takes at least several days. We have previously shown that there is excellent congruence between PFGE and MLST, with PFGE and MLST providing similar results for local epidemiological investigations[14].

MLST is being increasingly used to define the regional and global epidemiology of melioidosis[11,14,18] and has the benefit of absolute comparative ability across laboratories through the MLST website and unambiguous sequence type characterization. Nevertheless, MLST remains expensive and requires sequencing capability, thus currently restricting its availability for rapid determination of whether strains from a potential melioidosis outbreak are linked.

PCR-based methods lend themselves to obtaining rapid results. Although randomly amplified polymorphic DNA (RAPD) analysis has been used for *B. pseudomallei*[19], reproducibility between and even within laboratories is a major problem and despite its quick results we have abandoned using it[20]. Excellent results have recently been reported using a multiplex PCR-based multilocus variable-number tandem repeat (VNTR) assay to assess the 2004 upsurge of melioidosis in Singapore. Liu et al. were able to use a VNTR system developed from the *B. pseudomallei *genome data to demonstrate diversity rather than clonality amongst the *B. pseudomallei *strains isolated from the cluster of melioidosis cases[21].

In investigating case clusters of melioidosis in northern Australia we have used PFGE to link cases to water supply contamination[8] and to contamination of a container of detergent[10]. We have also shown that in our melioidosis endemic region case clusters during extreme weather events are usually not genetically linked by PFGE fingerprinting[6]. As in Singapore in 2004[21] these clusters are simply reflecting the close association between rainfall and infection from the diverse range of *B. pseudomallei *strains present in soil and surface water.

**Figure 3:**
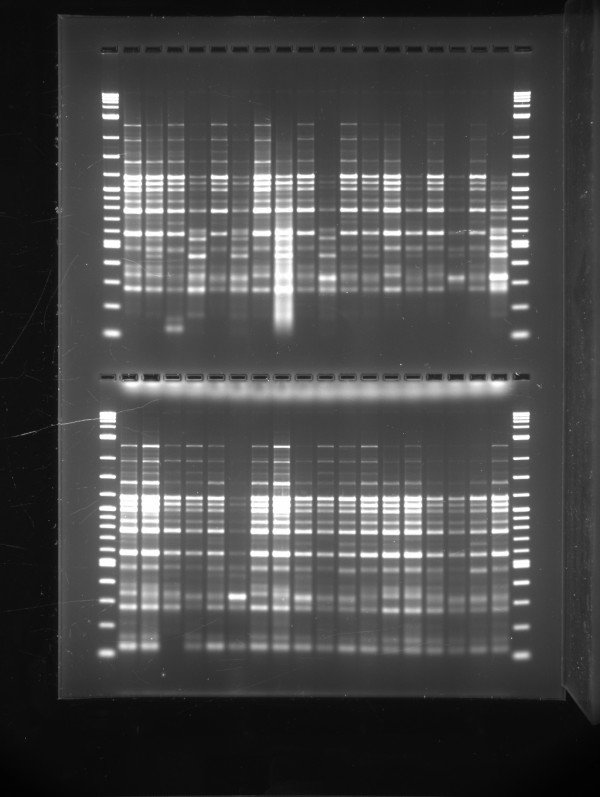
A BOX-PCR gel showing two sets of results, each with 18 isolates of *B. pseudomallei*, with molecular weight markers on the outside lanes.

Because PFGE takes up to 5 days for results, we are assessing alternative typing options for a rapid determination of whether a cluster of melioidosis cases is genetically linked and therefore potentially an outbreak which requires an urgent public health response. BOX-PCR typing can be completed within 1 working day and has shown generally good agreement with PFGE for *B. cenocepacia*[9]. We have now demonstrated that BOX-PCR can perform similarly for *B. pseudomallei*, with ability to usually discriminate between non-clonal isolates, while also showing relatedness within clonal groups. An important issue for BOX-PCR is that with current methods it can be less reproducible than PFGE[9]. We have found variations when comparing results from different PCR machines in our laboratory and in addition the band density differentials are DNA template concentration dependent (data not shown). These issues currently preclude reliable comparisons of BOX-PCR results between laboratories. In addition, while BOX-PCR gels can be visualised directly for assessing small numbers of isolates (Figure 3), our methodology for electronic analysis of larger isolate numbers requires purchase of software modules.

## Conclusion

By including in a single run a number of reference strains known to show considerable diversity on BOX-PCR our data suggest that BOX-PCR can be used to exclude a clonal outbreak of melioidosis within 10 hours of receiving the bacterial strains. Subsequently, MLST can show the relatedness of an outbreak strain to other isolates by querying the MLST database.

## Competing interests

The author(s) declare that they have no competing interests.

## Authors' contributions

BJC conceived the study and drafted the manuscript. DG and MM carried out the PFGE and BOX-PCR, DG the MLST and LW helped analyse the comparisons. JJL and BGS provided expertise on methodology and interpretation of the typing methods and also helped draft the manuscript. All authors read and approved the final manuscript. The authors have no conflicts of interest.

## Pre-publication history

The pre-publication history for this paper can be accessed here:


